# Neonatal Hypoxic-Ischemic Encephalopathy Yields Permanent Deficits in Learning Acquisition: A Preclinical Touchscreen Assessment

**DOI:** 10.3389/fped.2020.00289

**Published:** 2020-06-05

**Authors:** Jessie R. Maxwell, Amber J. Zimmerman, Nathaniel Pavlik, Jessie C. Newville, Katherine Carlin, Shenandoah Robinson, Jonathan L. Brigman, Frances J. Northington, Lauren L. Jantzie

**Affiliations:** ^1^Department of Pediatrics, University of New Mexico, Albuquerque, NM, United States; ^2^Department of Neurosciences, University of New Mexico, Albuquerque, NM, United States; ^3^Division of Neonatal-Perinatal Medicine, Department of Pediatrics, Johns Hopkins University School of Medicine, Baltimore, MD, United States; ^4^Department of Neurosurgery, Johns Hopkins University School of Medicine, Baltimore, MD, United States; ^5^Department of Neurology, Johns Hopkins University School of Medicine, Baltimore, MD, United States; ^6^Department of Neurology, Kennedy Krieger Institute, Baltimore, MD, United States

**Keywords:** biobehavioral biomarker, HIE, touchscreen, learning acquisition, cognitive flexibility, reversal learning

## Abstract

Neonatal hypoxic-ischemic encephalopathy (HIE) remains a common problem world-wide for infants born at term. The impact of HIE on long-term outcomes, especially into adulthood, is not well-described. To facilitate identification of biobehavioral biomarkers utilizing a translational platform, we sought to investigate the impact of HIE on executive function and cognitive outcomes into adulthood utilizing a murine model of HIE. HIE mice (unilateral common carotid artery occlusion to induce ischemia, followed by hypoxia with a FiO_2_ of 0.08 for 45 min) and control mice were tested on discrimination and reversal touchscreen tasks (using their noses) shown to be sensitive to loss of basal ganglia or cortical function, respectively. We hypothesized that the HIE injury would result in deficits in reversal learning, revealing complex cognitive and executive functioning impairments. Following HIE, mice had a mild discrimination impairment as measured by incorrect responses but were able to learn the paradigm to similar levels as controls. During reversal, HIE mice required significantly more total trials, errors and correction trials across the paradigm. Analysis of specific stages showed that reversal impairments in HIE were driven by significant increases in all measured parameters during the late learning, striatal-mediated portion of the task. Together, these results support the concept that HIE occurring during the neonatal period results in abnormal neurodevelopment that persists into adulthood, which can impact efficient associated learning. Further, these data show that utilization of an established model of HIE coupled with touchscreen learning provides valuable information for screening therapeutic interventions that could mitigate these deficits to improve the long-term outcomes of this vulnerable population.

## Introduction

Neonatal hypoxic-ischemic encephalopathy (HIE) occurs in as many as 6 infants per 1,000 live births (1–4). While there is likely an acute event occurring around delivery, the injury observed in HIE is likely a combination of acute on chronic injury (5). Recent placental studies have found that chronic fetal vascular malperfusion appears to be associated with neonatal HIE, leading to the hypothesis that impaired fetal blood flow may result in the infant being more susceptible to brain injury from altered cerebral blood flow in the setting of an acute event (5, 6).

Children with a history of HIE are at high risk of abnormal cognitive development. Unfortunately, more than half of infants have abnormal neurodevelopment following HIE (1, 7). Additionally, even those infants with mild HIE, previously thought to have normal outcomes, have been observed to have abnormal neurodevelopment with deficits apparent in childhood including lower cognitive composite scores (1, 8). While executive function is an umbrella term used to describe the processes necessary to achieve goal-directed behavior, multiple domain specific deficits have been reported after HIE in children. A recent analysis reports that 22% of children with moderate HIE and no cerebral palsy were found to attend special education, compared to all of the control group attending mainstream school (9). Thus, it is paramount that we further investigate the long-term deficits in cognition these children may have following this perinatal injury.

Characterizing the spectrum of brain injury and identifying biobehavioral biomarkers are critical to predict outcomes and advance novel therapeutic interventions for children with HIE. Brain magnetic resonance imaging (MRI) was used in a term infant population with neonatal encephalopathy, and found evidence of acute insult without evidence of established injury or atrophy in 69–80% of the infants imaged (10–12), supporting the concept that the brain injury is occurring around the time of delivery. Specific regions of the brain seem to be more susceptible to injury in the setting of HIE, and include cerebral cortex, basal ganglia, putamen, thalamus, and brainstem (13–15). Additionally, cerebral white matter is also often injured (15) as well as the hippocampus (16).

Animal models have been utilized to characterize the brain injury following HIE more specifically, with rigorous characterization of the pathophysiology occurring during the newborn period. The Rice-Vannucci model is currently accepted as the standard model of term HIE. Studies have described the injury that occurs following this model, including cellular injury from mitochondrial dysfunction and oxidative stress, injury to the hippocampus, caudate-putamen, cerebral cortex, and thalamus (17–20). Currently, data defining the specific pillars of cognition impacted in adulthood by HIE is a gap in knowledge. Therefore, we sought to test the hypothesis that HIE would yield deficits in complex cognitive and executive functioning using a translational touchscreen approach sensitive enough to detect changes in functional outcome across many models of perinatal brain injury. Specifically, we tested the effects of neonatal HIE on a touchscreen platform to assess cognitive function in adulthood and we examined whether pairwise visual discrimination learning and reversal, known to be mediated by separate regions of the brain (21), were sensitive to long-term injury following HIE. Utilizing a touchscreen platform that incorporates stimuli, learning rules and response actions that mimic those used in human cognitive assessments such as the Cambridge Automated Neuropsychological Test Automated Battery (CANTAB), we investigated a validated, translational measure of cognitive function following HIE (22–24). Discrimination and reversal learning are heavily dependent on orbitofrontal-striatal connections in both humans and rodents (25, 26). Reversal learning is a paradigm to assess cognitive flexibility in the setting of changing stimulus-outcome or response-outcome (26). Different stages of learning can be assessed by looking at specific times during the reversal paradigm, including early (<50% correct) and late (>50% correct). Thus, the utilization of this testing paradigm allows for insight into specific characterization of cognitive deficits observed followed HIE in a preclinical model. We predicted that mice subject to HIE would have cognitive deficits as adults, defined by deficits in reversal learning, specifically the learning or later portion, reflective of the structural brain injury extensively published in this model (13, 17–20, 27–32).

## Materials and Methods

### Animals

All experimental procedures were approved by the Institutional Animal Care and Use Committee (IACUC) of the University of New Mexico. All animal studies were carried out with standards of care and housing in accordance with the National Institutes of Health Guide for the Care and Use of Laboratory Animals, US Department of Health and Human Services. C57BL/6 male (*n* = 12) and female mice (*n* = 16) were used for a total of 28 mice.

### Neonatal Hypoxic Ischemic Brain Injury Model

To induce hypoxic-ischemic (HI) injury in a term-equivalent population, the Rice-Vannucci model was adapted for use in mice at postnatal day 10 (P10), as previously published (18, 29, 33–35). Briefly, mice were anesthetized with 3% isoflurane for induction and maintained with 1% isoflurane throughout the procedure and kept normothermic. The right common carotid artery was isolated and ligated via double suture to result in a permanent unilateral carotid ligation. The incision was closed with dermabond, and the mouse recovered for 1 h with their littermates. Mice with unilateral carotid ligation then underwent hypoxia for 45 min with an FiO_2_ of 0.08 in a humidified, temperature-controlled hypoxia chamber (29, 36, 37). Sham mice were anesthetized, with carotid artery isolated but not ligated. They were not exposed to hypoxia. Each litter consisted of both HI and sham mice. Following completion of the experiment, mice matured with their dams until weaning at P21.

### Touchscreen Cognitive Assessment

All operant behavior was conducted in a chamber measuring 21.6 × 17.8 × 12.7 cm (model # ENV-307W, Med Associates, St. Albans, VT) housed within a sound- and light attenuating box (Med Associates, St. Albans, VT). A solid acrylic plate was used to cover the grid floor of the chamber to facilitate ambulation. A peristaltic pump delivered 30 μl of liquid strawberry milkshake (strawberry Nesquik mixed with skim milk) into a magazine as required. A house-light, tone generator and an ultra-sensitive lever was located on one end of the chamber, while a touch-sensitive screen (Conclusive Solutions, Sawbridgeworth, U.K.) was on the opposite side of the chamber covered by a black acrylic aperture plate, which creates two 7.5 × 7.5 cm touch areas separated by 1 cm and located at a height of 0.8 cm from the floor of the chamber. KLimbic Software Package v1.20.2 (Conclusive Solutions) controlled and recorded stimulus presentation and touches in the response windows.

#### Pretraining

Beginning at 8 weeks of age (~P60 or adolescent human age equivalent), all mice were handled daily and were food-restricted to 85% of their free-feeding body weight. Food restriction ensures animals are properly motivated to obtain the reward during task performance and did not begin until all mice had reached 20 grams in weight. While HI mice were slightly smaller after HI surgery and through the second and third postnatal week consistent with the model, all mice reached the same weight prior to starting the food restriction. Operant training began once mice reached food-restricted weight at ~10 weeks of age. Mice were first acclimated to the liquid reward by provision of ~30 μl/mouse in the home cage for 3 days and then habituated to retrieving reward in the operant chamber. Mice were allowed 30 min to freely retrieve rewards available in the magazine. Mice retrieving at least 10 rewards within 30 min were moved to lever press training. Here, mice could only obtain reward by responding on an ultrasensitive lever within the chamber. Reward delivery was accompanied by the presentation of secondary reinforcers: a 2-sec, 65 dB auditory tone and illumination of a magazine light. For each trial, mice were required to collect the delivered reward (measured by magazine beam-break) before another reward was available via an active lever response. Mice were required to lever-press and collect 30 rewards in under 30 min before moving to acquisition testing.

#### Discrimination and Reversal

**Figure 1:**
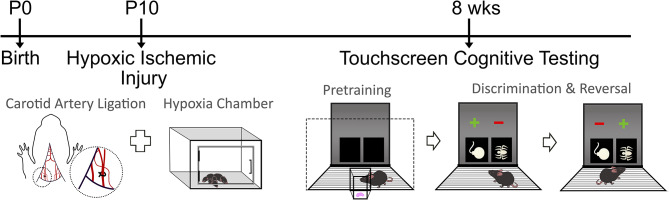
Timeline of experimental design. Mice randomized to the HIE group underwent the carotid artery ligation and hypoxia chamber exposure at postnatal day 10 (P10). Following recovery, the touchscreen cognitive testing started with pretraining, followed by discrimination and reversal tasks in both sham and HIE groups. During pretraining, mice became familiar with the chamber and the food reward system. In discrimination, one symbol was the correct answer, which would result in a food reward if chosen. During reversal, the symbol that was previously correct became the incorrect response.

Pairwise discrimination and reversal was tested as previously described (38–41). Briefly, mice were first trained to discriminate 2 novel, approximately equally-luminescent stimuli, presented in the center of each window in a spatially pseudorandomized (left/right) manner, over 30-trial sessions (5-s inter-trial interval) using their nose (21, 42). The stimulus designated as correct was counterbalanced across mice and treatment. Responses at the correct stimulus window resulted in a 30 μl liquid reward, cued by a 1-s tone and illumination of the magazine. Responses at the incorrect stimulus window resulted in a forced timeout (10 s), signaled by illumination of the house-light. Correction trials following errors were presented with the correct stimulus presentation in the same window until a correct response was made. Discrimination criterion was ≥85% correct responding out of 30 trials, excluding correction trials, over 2 consecutive sessions. Reversal training began on the session after discrimination criterion was attained. Here, the designation of correct verses incorrect stimuli was reversed for each mouse. As for discrimination, there were 30-trial daily sessions until the mice reached a criterion of ≥85% correct responding (excluding correction trials) over 2 consecutive sessions. Figure 1 summarizes the surgical procedure completed at P10, followed by recovery and pretraining on the touchscreen platform at 8 weeks of age. Visual discrimination was then completed, in which one stimulus was the correct response. Upon reaching criterion, reversal testing occurred in which the previously correct stimulus was now incorrect.

### Statistical Analysis

For discrimination and reversal, the following dependent variables were analyzed: trials, errors, correction errors, reaction time (time from lever press initiation to screen touch) and magazine latency (time from screen touch to reward retrieval). In order to examine distinct phases of reversal (early perseverative and late learning) errors and correction errors for sessions where performance was below 50% and performance from 50% to criterion, were separately analyzed as previously described (25, 43). To analyze use of feedback for learning, correct and incorrect responses were further categorized based on previous trial outcome: correct responses were characterized as *win-stay* (following correct response) or *lose-shift* (following an error trial), while error trials were characterized as *perseverative* (following an error trial) or *regressive* (following correct response) as previously described (40). As assumptions for normality and equivalent variance were met, data were analyzed using unpaired *t*-tests followed by Bonferroni correction for multiple comparisons. Data is represented as mean ± standard error of the mean (SEM), with *p* < 0.05 designated as statistically significant.

## Results

**Figure 2:**
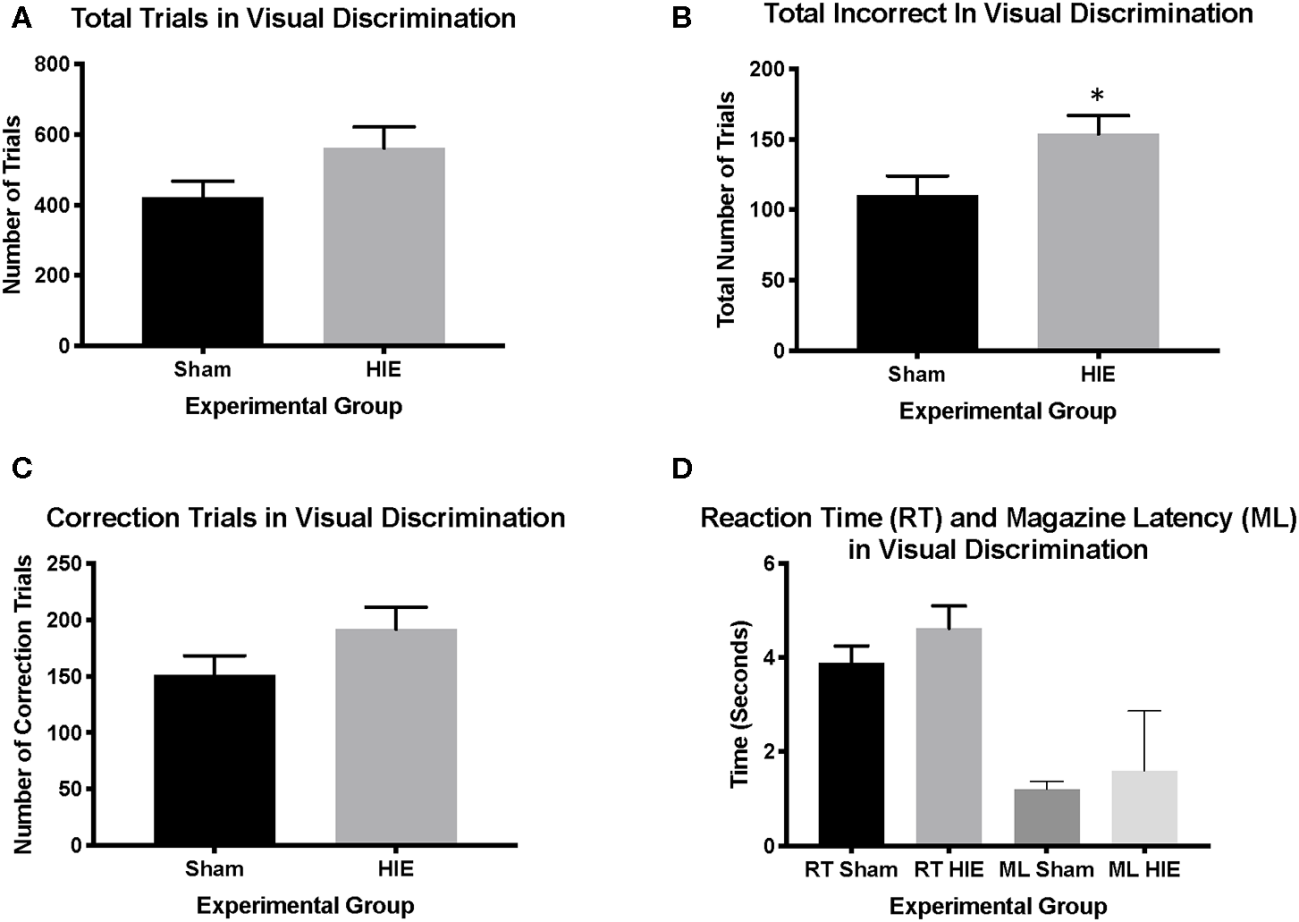
Visual discrimination revealed more incorrect trials in the HIE group compared to sham. During visual discrimination, the total number of trials between the sham and the HIE group were not significantly different **(A)**. The HIE group did require significantly more incorrect trials to complete the visual discrimination component compared to sham **(B)**. Although the HIE group had more correction trials during visual discrimination, this was not significant **(C)**. There was no difference in either the reaction time or magazine latency between the two groups during visual discrimination **(D)** (*n* = 13–15, *p < 0.05). Data is represented as mean ± standard error of the mean (SEM).

Analysis of sex as a biological variable found no main effect of sex on any measure, and thus both sexes were combined by treatment for subsequent analysis [12 males (6 sham, 6 HIE) and 16 females (7 sham and 9 HIE) were used in total]. The average weights of the mice prior to food restriction was 20.01 grams ± 0.79. Examining pretraining, we found no significant differences between groups on any of the three stages (HIE: *n* = 15; Sham: *n* = 13). All mice in each group successfully completed visual discrimination to criterion. However, while the total number of trials during visual discrimination did not differ significantly between the HIE and sham groups (*t*_(26)_ = 1.768, *p* = 0.09; Figure 2A), the HIE group had significantly more incorrect responses compared to sham (*t*_(26)_ = 2.304, *p* = 0.03; Figure 2B). Additionally, no significant difference was noted in the number of correction trials during visual discrimination (*t*_(26)_ = 1.540, *p* = 0.13; Figure 2C). There was also no significant difference in the reaction time (*t*_(26)_ = 1.204, *p* = 0.24) or magazine latency (*t*_(26)_ = 1.114. *p* = 0.28) between the two groups during visual discrimination (Figure 2D), suggesting that alterations in learning were not due to differences in motor behavior or motivation to work for reward.

**Figure 3:**
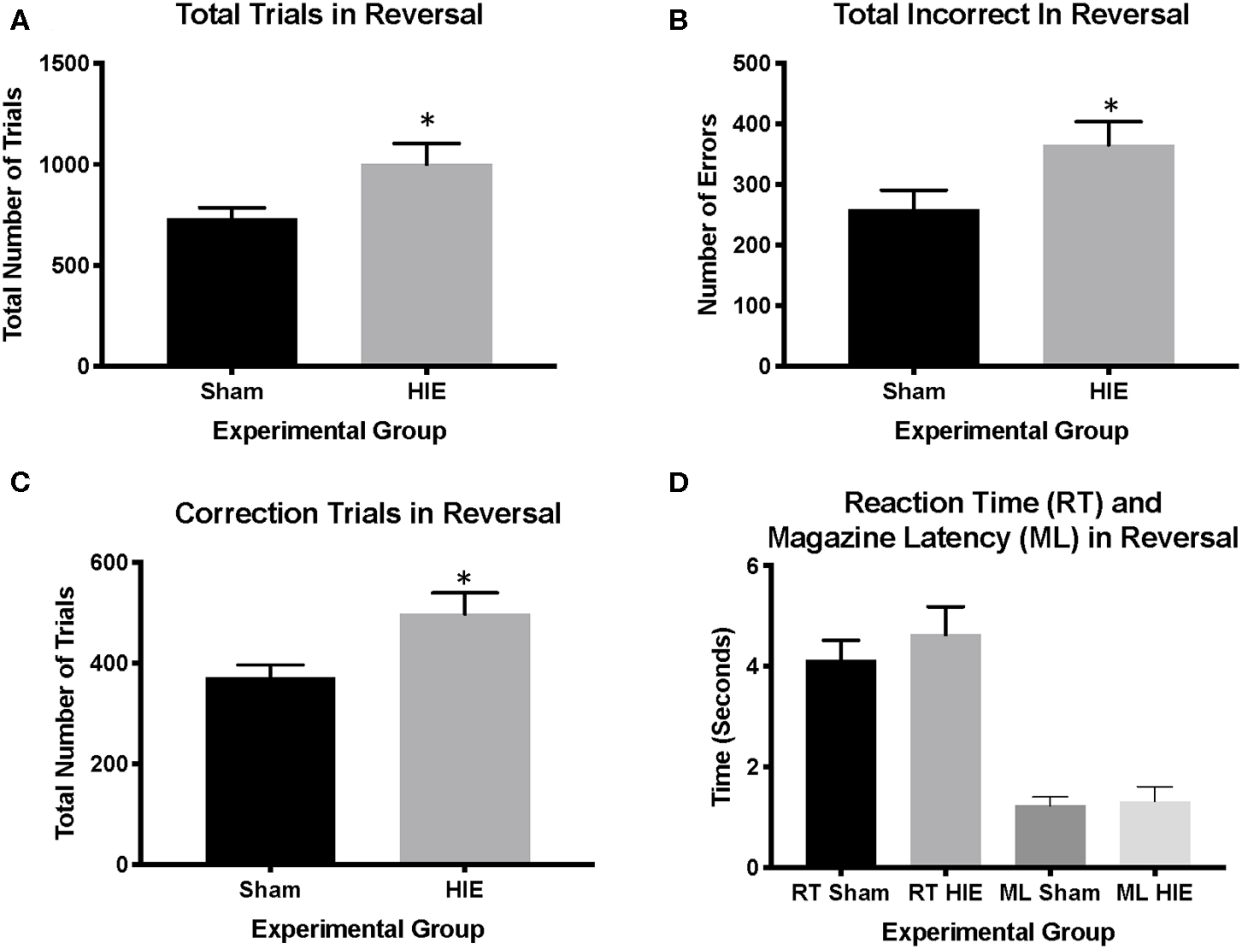
HIE induces reversal learning Deficits. The HIE group required significantly more trials to complete reversal compared to the sham group **(A)**, as well as more incorrect responses during reversal **(B)**. Additionally, the HIE group completed significantly more correction trials compared to the sham group **(C)**. There was no difference in either the reaction time or magazine latency between the two groups during reversal **(D)** (*n* = 13–15, *p < 0.05). Data is represented as mean ± standard error of the mean (SEM).

**Figure 4:**
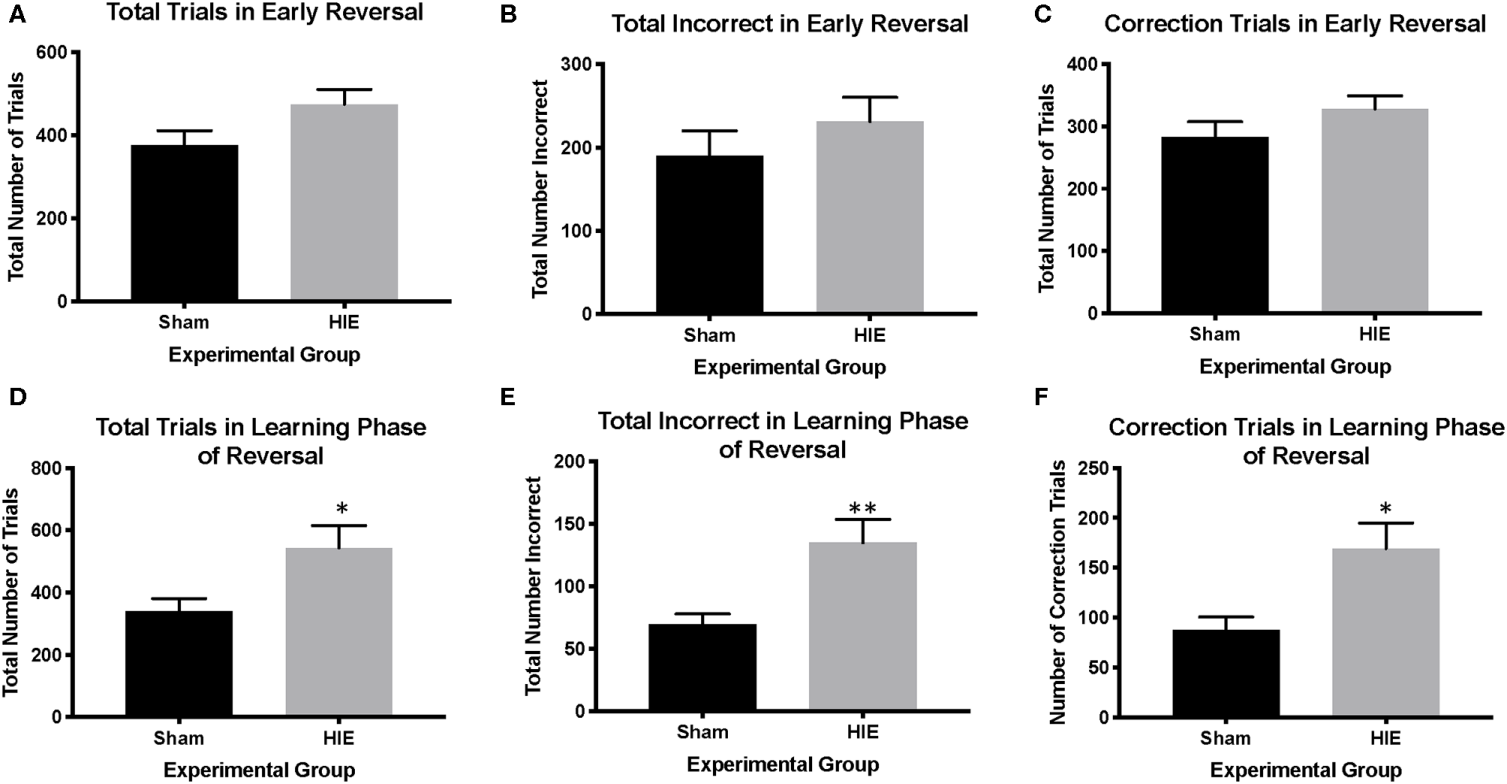
HIE yields distinct early phase and learning phase reversal deficits. In the early phase of reversal, there were no significant differences between HIE and sham in the total trials completed, the total incorrect, and the number of correction trials **(A–C)**. However, in the learning phase of reversal, the HIE group had significantly more total trials **(D)**, more incorrect trials **(E)** and more correction trials **(F)** compared to sham (*n* = 13–15, *p < 0.05, **p < 0.01). Data is represented as mean ± standard error of the mean (SEM).

Analysis of reversal performance revealed multiple significant differences between HIE and Sham control animals. HIE mice required significantly more total trials to complete the reversal compared to sham (*t*_(26)_ = 2.196, *p* = 0.02, Figure 3A). Similarly, HIE made significantly more errors (*t*_(26)_ = 2.118, *p* = 0.04, Figure 3B) and correction errors (*t*_(26)_ = 2.494, *p* = 0.02, Figures 2, 3C) compared to sham control animals. Consistent with discrimination, HIE and sham did not differ significantly on either reaction time (*t*_(26)_ = 0.911, p=0.37) or magazine latency (*t*_(26)_ = 0.722, *p* = 0.48) across the reversal (Figure 3D). Analysis of reversal learning by stage revealed that HIE animals needed similar number of trials (*t*_(26)_ = 1.948, *p* = 0.06) and made similar numbers errors (*t*_(26)_ = 0.978, *p* = 0.33) and correction errors (*t*_(26)_ = 1.384, *p* = 0.18) during the early, perseverative, stage of reversal (Figures 4A–C). However, during the learning phase of reversal testing, the HIE group needed significantly more trials (*t*_(26)_ = 2.374, *p* = 0.03, Figure 4D), and made significantly more errors (*t*_(26)_ = 3.021, *p* = 0.005, Figure 4E) and required more correction trials (*t*_(26)_ = 2.723, *p* = 0.01; Figure 4F) vs. controls. While both the HIE and control group were able to complete the visual discrimination portion of the paradigm, the HIE group had more significant differences during the late, learning phase of reversal, a portion of testing sensitive to striatal-mediated functions.

## Discussion

While clinical studies have shown significant cognitive delays at 18 to 24 months of age following HIE as determined by lower mental developmental index scores and subnormal intelligence quotient scores at age 6 to 7, outcome studies beyond this time are extremely limited (44). Here, we investigated the long-term impact of HIE on learning and cognitive flexibility in adult mice following a neonatal HIE insult utilizing a translational touchscreen task. We found that HIE at term equivalent stage of development was sufficient to produce permanent deficits in learning, both in discrimination and late stage reversal learning. To date, this is the first report of deficits of cognition using a translational touchscreen platform and the first evidence establishing the long-term cognitive deficits that can occur following HIE in rodents. Prior studies in animals however have focused on sensory motor impairments and basic assessments of reflexes. For example, Y-maze testing and object location task after HIE, without and with therapeutic hypothermia, revealed a lower exploratory preference after HIE. The memory impairments were not altered by therapeutic hypothermia (33). In a different study, three developmental reflexes (righting, cliff aversion and geotaxis) were assessed 24 h after HIE in mice, and correlated with Morris Water Maze testing 8 weeks later (45). The Morris Water Maze testing of navigational learning and memory supported neurofunction deficits, leading the authors to conclude that sensorimotor reflex performance in the acute phase of HIE may have predictive values for long-term outcome (45). Given that these tests rely on aversive motivation, we chose to utilize the touchscreen testing platform which relies on positive motivation (food) as a means to test the hypothesis that HIE yield complex impairments in cognition and executive function that are specific to brain regions susceptible to injury. Additionally, touchscreen offers sophisticated, reproducible analysis of multiple pillars of behavior using a platform that directly corresponds to human testing (46–48).

Children with a history of HIE are at high risk of abnormal cognitive development. However, few studies have utilized animal models of HIE to examine the long-term impacts on cognitive function, with testing occurring into adulthood. Notably, mice at the conclusion of touchscreen training are over 150 days old. Utilizing a touchscreen platform, we found multiple deficits in learning, both during an initial pairwise discrimination, and during late stage reversal, when animals learn the new response rule to a high degree of consistency. HIE did not globally impair response learning, as mice were able to learn initiation and response behaviors in the absence of a discrimination during pretraining at levels similar to control. During discrimination, HIE animals showed consistently lower performance on all measures, although they were only significantly worse than control as measured by first-presentation correction errors. Importantly, secondary latency measures, such as reaction time and the time to retrieve a reward, did not differ across groups, suggesting that HIE did not globally impair motor behavior, or motivation to work for food reward. While they did have more incorrect responses on their way to completing the discrimination task, all HIE mice were able to perform at a high criterion level, allowing us to test them on reversal learning.

During reversal, the HIE group demonstrated a global impairment as measured by number of trials, errors and correction errors across the entire paradigm. However, the impairment was not typified by a cortical-mediated loss of flexibility. Reversal learning, and particularly the early, perseverative phase, has consistently been shown to be mediated by the orbitofrontal cortex (OFC) across species (26, 49–52). In the current study, HIE did not significantly differ on any measure during the perseverative phase (39, 40, 53). Rather, analysis of learning stage revealed that the reversal impairment was driven by significant increases in trials, errors, and correction trials during the later stages of reversal, when performance has reached or exceeded chance levels. Together, the increase in incorrect responses during initial learning together with impaired late-stage reversal suggest an impairment in the acquisition of well-trained stimulus-response contingencies, which is mediated by subcortical structures such as the dorsal striatum (dS) (25, 39). The efficient balance of associative learning and behavioral flexibility is mediated by cortico-striatal-thalamic loops (54) and single unit recording and imaging studies have demonstrated that the striatum plays a critical role in the representation of reward-action relationships required to efficiently guide choices (25, 55, 56). Interestingly, the touchscreen results of impaired learning implicate the dorsal striatum, in alignment with evidence that human infants with term HIE are more likely to have injury to the basal ganglia on present on neuroimaging evaluation.

Our study provides evidence of a significant and selective deficits in associative learning in mice with HIE. Upon acquiring a pairwise visual discrimination, when the reinforcement contingencies of the learned association were reversed, HIE mice were significantly impaired compared to shams. This was due to deficient learning of the new association rather than impaired reversal *per se*. Both groups performed at equivalent levels during reversal sessions when performance was low and perseveration high (i.e., <50%), while HIE mice committed more trials and errors during sessions when performance was largely learning related (i.e., >50%). This deficit is in contrast to previously published touchscreen assessments in animal models of perinatal brain injury (38, 41, 57). Specifically, adult rats perinatal brain injury secondary to chorioamnionitis have a perseverative phenotype and significant deficit in cognitive control defined by a reversal deficit (38). Similarly, adult rats suffering severe traumatic brain injury in infancy perseverate, lack cognitive flexibility and struggle to pass reversal learning criteria (41). The extensive white matter brain injury and orbitofrontocortical decoupling observed in both chorioamnionitis and traumatic brain injury may partially explain these findings. Interestingly, animals with perinatal exposure to methadone have a mixed phenotype of executive dysfunction with rats committing significantly more correction errors both during the perseverative phase and during the later learning phase compared to saline control animals (57). Together, these data indicate that adult rats exposed to perinatal methadone are impaired in both early and late reversal learning, consistent with global learning and executive control dysfunction (57) and widespread structural brain injury. In contrast to methadone exposure, prenatal alcohol exposure in mice results in behavioral inflexibility during early reversal testing, with persistent aberrant lateral orbital frontal cortex to dorsolateral striatum signaling (58). Taken together, these data indicate that the timing of injury (prenatal, perinatal or postnatal) has an impact on the type of cognitive deficits observed. While there are similarities in the injuries discussed such as inflammation, each injury results in a distinct behavioral phenotype. Notably, impaired performance as observed in HIE mice are not due to non-specific motivation or sensorimotor related performance as evidenced by normal scores on reaction response times and reward retrieval latency. Thus, although each injury model was different, the touchscreen platform is sensitive enough to detect distinct differences in cognitive domains following different types of perinatal brain injury.

Importantly, the impairments in learning during discrimination and reversal seen after HIE persist into adulthood. While more studies have reviewed outcomes of infants at a young age, few have observed this cohort into adolescence or adulthood (16). There has been greater recognition that HIE can have long-lasting impact on developmental trajectories, and studies have begun to assess the neurodevelopmental outcomes of children following HIE in the newborn period (9, 44, 59). Interestingly, children diagnosed with severe neonatal encephalopathy were found to be more than one grade behind the expected level for their age, with children diagnosed with moderate neonatal encephalopathy having difficulties in reading, spelling and mathematics (16). A recent prospective case-control study in the United Kingdom reported significantly lower mean full-scale IQ at 6–8 years of age after an HIE event, when compared to controls (59). Children with HIE also have significant differences in verbal comprehension, perceptual reasoning, working memory and processing speed (59). Similarly, a systematic review of five published studies reported a higher proportion of cognitive impairments at school age in children with a history of HIE, specifically in the area of executive functioning (9). The current results underline the idea that following infants diagnosed with neonatal encephalopathy is critically important, as they are at high risk of not only having difficulties at school age, but also into adulthood.

This study had limitations. While our data indicate significant differences in the HIE group, there is known variability of injury that occurs with the use of the Rice-Vannucci model (60). This variability can be beneficial, as it mimics the variation of injury observed in infants following HIE. Additionally, no environmental enrichment was used in the study, which could impact the neurodevelopmental outcomes (61, 62). Both sexes were utilized in this study, which adds to the generalizability of the results. The touchscreen platform is more robust and less vulnerable to environmental confounders than other modes of testing such as the Morris Water Maze. The rigor and reproducibility of the touchscreen testing, with the sensitivity of the measures obtained, allows for differences in performance from therapeutic interventions to be readily detected. Correlating outcomes on this touchscreen assessment of diverse pillars of cognition with pathology, lesion size, and multi-modal high-resolution neuroimaging could be investigated as a future direction. Finally, we did not include treatment within this study, such as hypothermia. Investigation the effect of hypothermia on the cognitive impairment reported here would be an essential element for future investigation as hypothermia is the only approved treatment for HIE in humans.

In sum, this is the first report that HIE in rodents is sufficient to cause long-lasting impairments in basal-ganglia mediated learning processes. This approach provides an important model platform for studies trialing adjunctive therapies to improve outcomes after HIE. Future studies should examine whether therapeutic hypothermia with additional therapies can mitigate the specific cognitive deficits that occur following HIE.

## Data Availability Statement

The datasets generated for this study are available on request to the corresponding author.

## Ethics Statement

The animal study was reviewed and approved by Institutional Animal Care and Use Committee (IACUC) of the University of New Mexico.

## Author Contributions

LJ conceptualized the hypothesis, designed and supervised the experiments. JM, AZ, NP, JN, KC, and LJ performed the experiments. JM, SR, JB, FN and LJ interpreted the data. JM, AZ, SR, FN, and LJ wrote the manuscript. All authors contributed to manuscript revision and approved the final version.

## Conflict of Interest

The authors declare that the research was conducted in the absence of any commercial or financial relationships that could be construed as a potential conflict of interest.
